# Very Low-Dose Risperidone in First-Episode Psychosis: A Safe and Effective Way to Initiate Treatment

**DOI:** 10.1155/2011/631690

**Published:** 2011-02-07

**Authors:** Patrick D. McGorry, John Cocks, Paddy Power, Peter Burnett, Susy Harrigan, Tim Lambert

**Affiliations:** ^1^Orygen Youth Health Centre for Youth Mental Health, The University of Melbourne, 35 Poplar Road, Parkville, VIC 3052, Australia; ^2^Lambeth Early Onset Service, Stockwell, London SW97AA, UK

## Abstract

Patients experiencing a first psychotic episode have high rates of extrapyramidal symptoms (EPSs) when treated with the doses of neuroleptics used in multiepisode or chronic schizophrenia. There is some evidence that lower doses may be equally, if not more, effective but less toxic in this population. Here, we report the results of a biphasic open label trial designed to assess the efficacy, safety, and tolerability of low-dose (2–4 mg/day) risperidone treatment in a group of 96 first-episode nonaffective psychosis patients. At the end of the trial, 62% of patients met the response criteria although approximately 80% had achieved a response at some time during the study. Reports of EPS remained low, and there were no dystonic reactions. We conclude that even at a dose of 2 mg/day, risperidone was highly effective in reducing acute symptomatology in a real world sample of young first-episode psychosis patients.

## 1. Introduction

Evidence increasingly suggests that early intervention with antipsychotic medications has a positive effect on treatment response and outcomes in patients with schizophrenia [1]. In contrast, a delay in initiation of antipsychotic treatment is associated with slower and less significant symptomatic recovery and poorer overall outcomes [2, 3]. The first five years after onset appears to be the critical window where the greatest functional decline associated with the illness occurs, indicating that initiation of treatment during this period may be particularly beneficial [4]. The progressive functional decline during this period may reflect, among other factors, alterations in brain structure and volume associated with increasing duration of illness in schizophrenia [5–7]. Early intervention with antipsychotic medications and psychosocial care has been demonstrated to modify these outcomes over time [8, 9].

As well as altering neurotransmitter activity, certain antipsychotic agents have a potentially neuroprotective effect. For example, Lieberman and colleagues [10] have demonstrated that olanzapine (dose range 5–20 mg/day), but not haloperidol (dose range 2–20 mg/day), may lead to a reduction in grey matter loss over time. Furthermore, low-dose risperidone (mean dose 2.8 mg) has been associated with increased grey matter in the superior temporal gyrus and middle temporal gyrus of patients [11]. These observations have been confirmed and extended in a very recent study by Thompson and colleagues [12], who showed that olanzapine treatment also blocked the trajectory of grey matter loss over the first 2 years after the initial psychotic episode, while haloperidol treatment did not. While the mechanisms underlying this protective effect are unclear, these and other studies indicate the importance of the first episode of psychosis (FEP) as a medication intervention point. 

Considerable evidence now indicates that low-dose antipsychotic treatment during the first episode is associated with symptomatic and functional improvement. Symptomatic improvements have been described using low-dose haloperidol at 3.4 mg/day [13, 14], low-dose olanzapine at 9.1 mg/day [15], and risperidone at doses of less than 6 mg daily [16, 17]. Superiority has also been demonstrated for the use of low-dose risperidone (mean 3.3 mg) over haloperidol (mean 2.9 mg) in terms of prevention of relapse and time to relapse in first-episode patients [18]. The EUFEST study, which compared the effectiveness of low-dose haloperidol (1–4 mg/day) with that of low doses of either amisulpride (200–800 mg/day), olanzapine (5–20 mg/day), quetiapine (200–750 mg/day), or ziprasidone (40–160 mg/day), has shown that while all five agents gave a similar degree of symptomatic improvement, treatment with haloperidol was associated with a significantly higher risk of EPS, and of discontinuation of medication. Thus, low doses of these atypical agents appear to be effective as well as acceptable to FEP patients [19].

While these data suggest that assertive treatment of the illness with antipsychotics is warranted to prevent changes in both brain morphology and functional outcomes, there is a case for caution with antipsychotics during the first episode. In particular, FEP patients tend to experience higher rates of extrapyramidal symptoms (EPSs) when treated with neuroleptics at doses recommended for chronic patients [1, 13]. As evidence indicates that the occurrence of side effects is a significant contributor to medication noncompliance in schizophrenia [20], it is possible that the use of recommended doses of neuroleptics in first-episode patients may be associated with poorer compliance and clinical outcomes. 

The atypical antipsychotic risperidone is a preferred treatment in FEP patients due to its more favourable tolerability profile and decreased rate of EPS at therapeutic doses compared to typical antipsychotics [21]. Kopala and colleagues [22] reported that low-dose risperidone (2–4 mg) led to lower rates of EPS and improved clinical outcomes compared to those seen for patients receiving recommended or high doses (5–8 mg). Merlo and colleagues [23] further demonstrated that over an 8-week treatment phase, risperidone at 2 mg/day was equally as effective as at 4 mg/day in first-episode patients and was associated with significantly less impact on fine motor function, a highly sensitive measure of EPS. In relation to chronic outcomes, Schooler and colleagues [18] demonstrated that chronic low-dose (3.3 mg) risperidone treatment (median 192 days) was associated with greater clinical improvement, less relapse, and less frequent and severe EPS compared to low-dose haloperidol (mean dose 2.9 mg) in FEP. 

Despite the apparent superiority of low-dose medication in many first-episode patients, treatment response is nonuniform, with only a proportion of patients responding to low-dose neuroleptics. McEvoy and colleagues [13] proposed two alternatives for overcoming neuroleptic nonresponsiveness in FEP patients. These include maintaining the initial dose for a longer period or increasing the dose to a predetermined ceiling level. A further treatment alternative to improve low-dose responsiveness is lithium combination therapy. This strategy has been widely used in patients with chronic schizophrenia, although the evidence for any beneficial effect, particularly for patients without an affective component, remains inconclusive [24]. While somewhat uncommon in FEP, the technique of combining lithium and atypical antipsychotics has been widely utilised in patients with bipolar disorder with combinations resulting in greater clinical improvements for many patients [25]. Tohen and colleagues [26] demonstrated that in bipolar patients, atypical antipsychotic and lithium combination therapy in patients nonresponsive to lithium or valproate monotherapy led to significant improvements with few additional side effects. As lithium has neuroprotective and neurotrophic properties, it is possible that combination therapy with lithium may provide neuroprotection in early psychosis in a similar manner [27, 28]. This data indicates that lithium may be a useful adjunctive therapy with atypical antipsychotics by both improving responsiveness to antipsychotic treatment in the short term and reducing the neuroprogressive deterioration in the long term [29]. Because the rate of grey matter loss is greatest during the year following a first psychotic episode [12], the provision of neuroprotection is an important component of the early therapeutic strategy, and adjunctive treatment with neuroprotective agents such as lithium, omega-3 fatty acids [30, 31], or N-acetyl cysteine [32] is increasingly considered as valuable approach. 

The aim of this pilot study was to evaluate the responsiveness to an initial 4-week course of low-dose (LD) risperidone (2 mg/day) treatment in young patients presenting with FEP, and to explore the efficacy and tolerability of three alternate treatment strategies for patients who did not respond during this initial treatment period. These were (A) extending the treatment period with the initial dose, (B) increasing the dose to 3 or 4 mg/day, or (C) adding adjunctive lithium therapy to the initial dose. We aimed to determine whether continuing risperidone treatment at the same dose for a further 4-week period would allow a clinically significant response, or whether either augmenting the dose of risperidone or adding lithium as an adjunctive agent was more effective over the same time frame.

## 2. Method

### 2.1. Patients

This was an open label study conducted at the Early Psychosis Prevention and Intervention Centre (EPPIC), now part of the Orygen Youth Health Centre for Youth Mental Health in Parkville, Victoria, between January 1996 and June 1997. The EPPIC program offers specialised care to young adults experiencing their first psychotic episode [33]. Patients were included if they were between 15 and 30 years of age, were experiencing a first episode of psychosis, defined with a DSM-IV diagnosis of schizophrenia, schizophreniform disorder, schizoaffective disorder, delusional disorder, psychotic disorder not otherwise specified, or brief psychosis, had psychotic symptoms requiring antipsychotic treatment, and had given written informed consent (or that of a legal guardian) to participate. 

Excluded from the study were female patients who were lactating or pregnant, patients with a mood disorder with psychotic features, or patients with an organic mental disorder including toxic confusional states, patients who had received a significant dose (more than 3 consecutive doses) of an oral neuroleptic in the previous 3 weeks, or a depot neuroleptic in the previous 8 weeks, patients who had received any investigational drug in the previous 4 weeks, patients receiving concurrent antidepressant medication, and patients with any clinically significant organic disease (including patients known to be HIV positive).

### 2.2. Procedure

**Figure 1:**
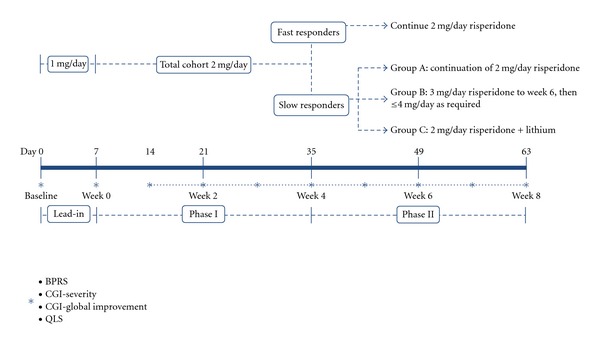
Timeline of the study.

The 8-week study involved two treatment phases (Figure 1). Primary efficacy parameters included change from baseline at the end of Phase I (Week 4) and Phase II (Week 8) on the Brief Psychiatric Rating Scale (BPRS) [34] and the Clinical Global Impression scale (CGI-severity and CGI-global improvement) [35]. Concomitant medication and adverse events were recorded at each assessment; and the patient's psychosocial functioning was evaluated over the course of the study using the Quality of Life Scale (QLS) [36].

During Phase I (Days 1–35, with Days 1–7 being the lead-in period), participants who were entered in the study completed baseline psychopathological assessments before or within 3 days of commencing risperidone at 1 mg/day. The dose of risperidone was increased to 2 mg on days 4–7, and weekly assessment commenced on day 14 (Figure 1). 

The therapeutic response at the end of Phase I (Week 4, day 35) was used to stratify treatment assignment for Phase II. Three measures were used to assess each participant's symptomatology and thus their response to treatment during Phase I: the BPRS-P, the CGI severity, and global improvement scales. Patients with a score of ≤3 on each of the BPRS psychosis subscale items (i.e., mild), a CGI (severity) rating of mild or less, and a CGI (global improvement) rating of at least minimally improved were considered “fast responders,” while all patients who did not meet these criteria were considered as “slow responders.” In Phase II (Weeks 4–8), fast responders continued on risperidone 2 mg/day, while slow responders were randomized single blind to one of three open treatment groups.

- Group A: continuation of risperidone 2 mg/day.
- Group B: dose increased to 3 mg/day for 2 weeks (Weeks 4–6), followed by a further increase up to a maximum of 4 mg/day (if required) for 2 weeks (Weeks 6–8).
- Group C: continuation of risperidone 2 mg/day with the addition of lithium titrated up to therapeutic levels (0.6–1.2 mmol) between days 35 and 42.

Clinical assessments were performed weekly for all patients during Phase II.

### 2.3. Statistical Analysis

Descriptive and inferential statistical procedures were performed to assess the characteristics of the sample and to examine the efficacy of the dosing strategies over the course of the study. Improvement on continuous psychopathology and clinical measures was assessed using paired samples *t*-tests. Comparisons between fast and slow responders at specific assessment points were undertaken using independent samples *t*-tests, and where the data were categorical, chi-square tests of significance were performed with exact tests performed where appropriate. Assumptions of parametric procedures were carefully evaluated prior to analysis and transformations applied to substantially skewed variables, such as duration of untreated psychosis (DUP). All statistical tests were two tailed and results regarded as statistically significant at or below the 5% probability level. A last-observation-carried-forward (LOCF) approach was adopted for analysis of Phase II data for all participants who completed Phase I.

## 3. Results

### 3.1. Treatment Efficacy

**Figure 2:**
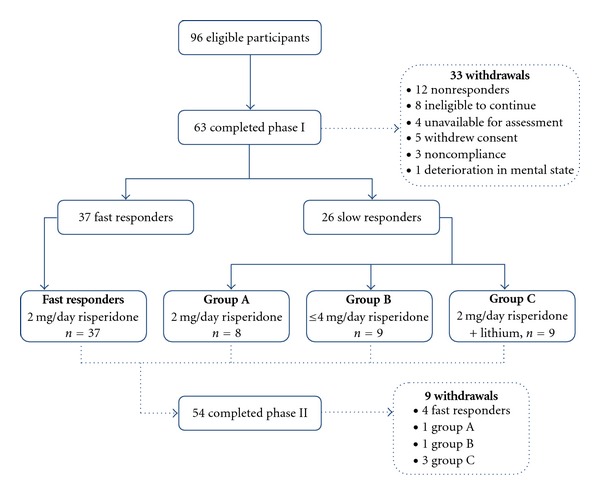
Disposition of patients over phases I and II of the study.

Of an initial sample of 96 participants, 63 (65.6%) remained in the study at the end of Phase I (Week 4). Thirty seven of the 63 participants (58.7%) were categorised as fast responders according to the study criteria, while 26 (41.3%) were slow responders (Figure 2). The sociodemographic characteristics of fast and slow responders as well as for those who withdrew in Phase I are compared in Table 1. Although all participants had received a DSM-IV diagnosis of a nonaffective psychotic disorder at entry, over the course of the study, nine participants had their diagnoses revised to that of an affective psychosis. Six of these nine participants were identified as having an affective psychosis during Phase I and hence were withdrawn from the study as ineligible to continue. However, for the other three participants, the diagnosis was not finalised until the end of Phase II. Because the study was conducted on an intention-to-treat basis, these three subjects were included in the Phase II analysis. Twenty-one participants were withdrawn due to protocol violations (ineligible to continue, not available for assessment, withdrew consent, noncompliant) and 12 due to a lack of response. There were no withdrawals due to adverse effects. At the end of Phase II (Week 8), 62% of the 63 patients had met the response criteria, with approximately 80% having responded at least once over the course of the study.

The mean change over time in psychopathology and outcome measures for all 63 participants and for the responder subgroups are shown in Table 2. In the total sample, there were significant improvements in the BPRS total and psychotic subscales and the CGI-S at week 4 (end of Phase 1) and week 8 (end of Phase II), with continued improvement during Phase II (Weeks 4–8). There was also a small but significant improvement from baseline in the BPRS negative symptoms subscale at week 4. This remained significant at the week 8 although there was no additional improvement during Phase II. Similarly, there was significant improvement in functioning as measured by the QLS at week 4. This remained significant at the end of Phase II without further improvement during Phase II. 

**Figure 3:**
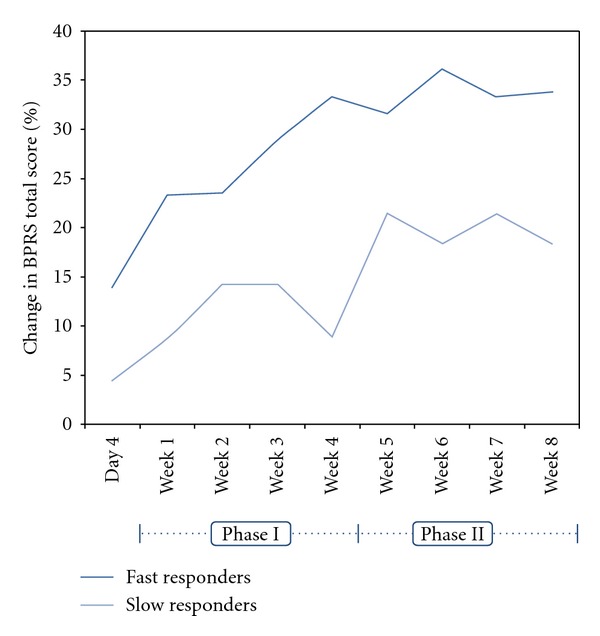
Percentage change in Brief Psychiatric Rating Scale (BPRS) total score from baseline for fast and slow responders during phase I (weeks 1–4) and phase II (weeks 4–8) of the study.

**Figure 4:**
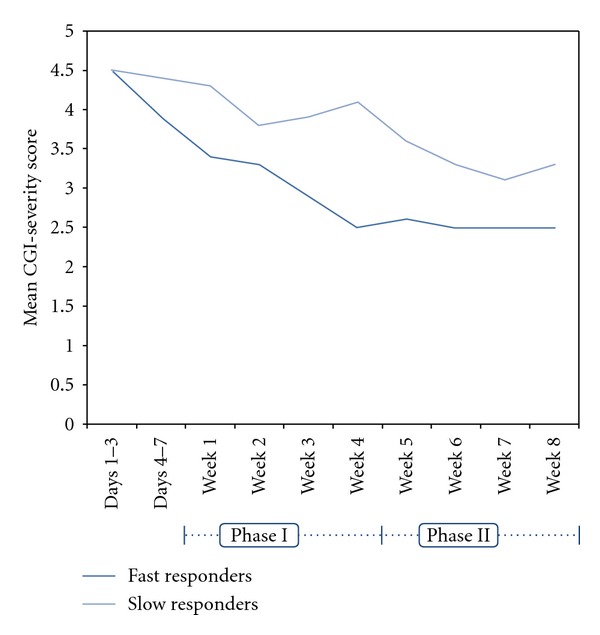
Comparison of the mean clinical global impression severity (CGI-S) scores for fast and slow responders during phase I (weeks 1–4) and phase II (weeks 4–8) of the study.

In terms of outcome variation between fast and slow responders, a different response pattern emerged. Fast responders tended to achieve significant improvement in psychotic symptoms by week 4, with little additional improvement during Phase II (Weeks 4–8). Slow responders continued to improve slowly over the 8 weeks (Table 2). There was, however, some fluctuation in response as indicated by the change from baseline in the BPRS total score at various time points in the study (Figure 3). Overall, fast responders tended to improve more than slow responders, with a tendency for greater improvements in symptom scores at the end of the study (Table 2, Figures 3 and 4). Also, only the fast responders had improvement in their psychosocial functioning, as measured by QLS. These improvements occurred during Phase I and were sustained during Phase II of the study.

While sample size restricted the use of statistical techniques, some variation was observed in the slow responders treated with different regimes. For example, the slow responders whose dose of risperidone was increased to 3 or 4 mg (Group B) experienced a greater symptomatic improvement on the BPRS at the end of Phase II than those in the low dose and combination therapy groups. Specifically, five of nine participants taking increased risperidone achieved an improvement equal to or greater than 20% on BPRS total scores and met the study criteria of a rating of ≤3 on each of the BPRS psychoses subscale items and a CGI (severity) rating of mild or less, for classification as responders. In contrast, one of nine patients in Group C (risperidone 2 mg + lithium) and no patients in Group A (risperidone 2 mg) achieved this result (exact *P* = .024). A nonsignificant result (exact *P* = .374) was observed for the response rates, with results at the completion of phase II indicating five of nine participants in group B, four of eight in group A, and two of nine participants in group C became classified as responders.

### 3.2. Adverse Events

Table 3 summarises the EPS reported during the study. Although 35% of patients experienced rigidity (usually mild) at least once over the course of the study, there were no reports of acute dystonia. Akathisia, again usually mild, was also reported at least once by 14% of the participants, and 16% reported mild tremor. Importantly, no symptoms were rated as severe, and it is notable that the increased doses of risperidone (mean 3.1 mg/day) in group B were not associated with any increased risk of EPS. 

In relation to weight gain, 66.1% of Phase I completers (*N* = 62; 1 missing) gained an average of 3.9 (SD = 2.7) kg in weight, 22.6% had no weight change, and 11.3% lost between 1.0 and 4.0 kg over the eight-week trial. The average weight change overall was 2.4 (SD = 3.1) kg with no significant differences between fast and slow responders. During Phase II, the average weight gain for the randomized slow responders was very similar: 1.0 kg in group A, 1.33 kg in group B, and 1.2 kg in group C.

Five participants experienced adverse events possibly associated with prolactin changes. Three of these events were reported during Phase I (two subjects reported sexual dysfunction and one reported gynecomastia at the week 2 visit) and two during Phase II (one participant reported irregular menstrual cycle at week 5, the other reported impotence at week 8).

## 4. Discussion

The primary aim of this study was to determine dosing strategies and sequences in young people with FEP. Previous studies have suggested that very low doses of both typical (haloperidol) and atypical neuroleptics may be sufficient to produce a response in acute episodes of schizophrenia, especially in FEP. The present study confirms previous demonstrations (e.g., [23]) that low-dose risperidone (2–4 mg/day) is an effective initial treatment choice in neuroleptic-naïve patients. The findings were consistent with previous research suggesting that 2 mg/day risperidone is an optimal initial dose in FEP patients [37]. Indeed, the higher than expected Phase I response rate (37 responders from 63 completers, or 58.7%) to 2 mg risperidone reduced the sample pool entering Phase II of the study. During Phase II, 4 of the 8 participants who continued on 2 mg/day risperidone met response criteria, bringing the overall response rate at this dose to 65%. Increasing the dose to 3-4 mg/day led to 5 of the 9 participants in this group meeting response criteria, an overall response rate of 73%. Zhang-Wong and coworkers [14] reported a 42% response rate with 2 mg of haloperidol in FEP, while higher dose risperidone (mean dose 6.1 mg) and haloperidol (mean dose 5.6 mg) led to 63% and 56% response in FEP patients, respectively [16]. In our study, a similar response rate as that observed by Emsley and colleagues [16] was achieved with one-third to half the dose of risperidone. This stepwise approach has the aim of reducing unnecessary adverse reactions, especially EPS.

Phase II of this study attempted to assess strategies for FEP patients who either did not respond, or responded more slowly, to low-dose risperidone. Unfortunately, due to the substantial withdrawal rate and the higher than expected response rate during Phase I, there was insufficient power to determine whether there were any significant differences in the relative efficacies of the different treatment strategies for slow responders. However, in clinical terms, a notable overall finding was that at the completion of Phase II, 62% of patients were considered to be responders, and approximately 80% had achieved a response at some point during the study. More specifically, during Phase II, 4 of the 8 participants assigned to group A (2 mg/day risperidone) met response criteria, while 5 of the 9 participants assigned to group B (3 or 4 mg/day risperidone), and 2 of the 9 participants in group C (2 mg/day risperidone + adjunctive lithium) also met response criteria. Thus, increasing the dose of risperidone to up to 4 mg/day may be beneficial for certain patients who do not show a rapid response to lower doses, since 55% of the initially nonresponsive participants who received this higher dose in our trial achieved a response without any associated increase in adverse events. While we were unable to determine the value of adjunctive lithium treatment in this trial, given the increasing recognition of the importance of neuroprotection as a treatment strategy in early psychosis, this approach merits further investigation in larger-scale trials.

The relatively low levels of EPS we observed are another important feature of this study. Mild rigidity was the most common symptom reported and was experienced at least once during the treatment phase by approximately one-third of participants. Mild akathisia and tremor were reported at least once by approximately one-sixth of participants. Most notably, no dystonic reactions were reported during the treatment phase, a major achievement in managing the initial treatment of FEP, especially given the good initial response rate. Our observations are in agreement with those of Kopala et al. [22], who also reported low levels of mild akathisia and no dystonia in FEP patients treated with low doses of risperidone. Similarly, Merlo et al. [23] reported low rates of EPS using doses of risperidone of 2 or 4 mg/day, although some cases of dystonia did occur, perhaps as a result of adjunctive typical antipsychotics that were used for sedation in some of the patients in the study. In contrast, in studies of FEP patients using recommended doses of conventional neuroleptics, 62% or more of FEP patients experience EPS [16, 38, 39]. Current recommended doses of risperidone are between 4 and 6 mg/day [40]; however, the results of the current study and all others indicate that this dose regimen is unwarranted in the majority of first-episode patients. Further, this dose range potentially exposes patients to a higher risk of EPS, thus negating the atypical neuroleptics' undoubted advantage of improved subjective tolerability. 

A final side effect of neuroleptic medications is weight gain. There has been particular concern that certain of the newer agents have a greater potential to induce weight gain. The overall average weight gain in this study of 2.4 kg over 8 weeks is consistent with a systematic analysis of published data [41] and real-world observational studies [42], indicating that this agent may be preferable to patients in this age group (i.e., 15–24 years). The weight gain we observed is less than that previously reported for olanzapine and clozapine, and possibly less than certain of the newer agents such as ziprazidone and aripiprazole.

The molecular mechanisms that underlie the variability in effectiveness of the antipsychotic drugs are still unknown. The therapeutic effects of low-dose risperidone may be a direct result of individual variations in the levels of dopamine receptor occupancy. Risperidone has a shallow dopamine occupancy curve, and thus at low doses a high serotonin-dopamine occupancy ratio may be present [43]. Data from positron emission tomography (PET) studies indicated that optimal dopamine D_2_ receptor occupancy was between 50% and 80% for maximal therapeutic effects and minimal EPS. However, D_2_ occupancy above 80% is associated with increased risks of EPS [44–46]. In first-episode patients, low doses of neuroleptics may lead to these targeted levels of receptor occupancy. For example, desirable D_2_ receptor occupancy levels of 66% have been reported with risperidone at 2 mg/day and 50% at 1 mg/day [47, 48]. However, high-dose risperidone (6 mg/day) has high levels of both receptor occupancy (82%) and EPS compared with lower doses. These data indicate that low-dose risperidone may not only be associated with better clinical improvements through its activity at dopamine receptors and to a lesser degree serotonin receptors, but low-dose regimens may also have neuroprotective effects through regulation of signalling pathways. This indicates that in first-episode psychosis, low-dose strategies (e.g., 2–4 mg/day risperidone) should be the benchmark for effective treatment in order to provide a balance between minimising symptoms and side effects while possibly providing neuroprotection for the patient in the form of altering signalling pathway activity in the brain. 

The limitations of this study include its small cohort and the significant dropout rate. This, combined with the unexpectedly high response rate in Phase I of the trial, meant that we were unable to determine the relative efficacies of the three alternative strategies proposed in Phase II for managing slow responders. Larger trials will be required to effectively address these issues. 

In conclusion, low-dose strategies improve the subjective quality of life of clients, thus improving their global treatment experience and potentially the therapeutic alliance established with their psychiatrist and other members of the clinical team. These findings of a better response to lower and hence safer doses of atypical antipsychotic medication in first-episode patients are consistent with the recently articulated model of clinical staging in psychiatry [49], which aims to refine diagnostic and treatment approaches to improve effectiveness and reduce harmful side effects for patients with mental disorders.

## Figures and Tables

**Table 1:** Sociodemographic and illness-related characteristics of the participants (*N* = 96).

	Fast responders *N = 37	Slow responders *N = 26	Withdrawn *N* = 33	*P* value
Sex (% male)	75.7%	69.2%	75.8%	.81

Mean age at admission, years (SD)	21.1 (3.7)	21.6 (4.0)	21.8 (3.4)	.75

Age at onset of psychotic symptoms, years (SD)	20.5 (3.5)	21.0 (3.8)	21.1 (3.5)	.77

Marital status (% never married)	81.1%	80.8%	90.9%	.44

Education (% post-secondary)	13.5%	19.2%	18.8%	.79

Diagnosis:				
Schizophrenia/schizophreniform	33 (89.2%)	19 (73.1%)	22 (66.7%)	
Schizoaffective/delusional/NOS	4 (10.8%)	4 (15.4%)	5 (15.2%)	
Affective disorder	0 (0%)	3 (11.5%)	6 (18.2%)	.094

Duration of untreated psychosis mean, (SD) and median (*M*) days	225.2 (269.8) *M* = 112.0	267.1 (416.5) *M* = 95.0	247.5 (370.5) *M* = 151.0	.97

*Responder status determined after 4 weeks of risperidone at a dose of 2 mg/day.

**Table 2:** Psychopathology and clinical improvement measures at baseline, week 4 (end of Phase I) and week 8 (end of Phase II).

Measure, mean (SD)		Total sample *N* = 63	Fast responders *N = 37	Slow responders *N = 26
BPRS total	*Baseline*	54.8 (9.2)	55.1 (9.5)	54.4 (8.9)
	*Week 4*	41.4 (9.8)^a^	35.9 (5.0)^a^	49.2 (9.6)^a^
	*Week 8^†^*	39.1 (9.3)^b,c^	35.7 (6.9)^c^	43.9 (10.3)^b,c^

BPRS psychotic subscale	*Baseline*	14.6 (2.9)	14.3 (2.9)	15.0 (2.8)
	*Week 4*	9.9 (4.0)^a^	7.3 (1.4)^a^	13.7 (3.4)
	*Week 8^†^*	8.6 (3.5)^b,c^	7.4 (2.6)^c^	10.4 (3.9)^b,c^

BPRS negative symptoms subscale	*Baseline*	6.3 (2.2)	6.1 (2.2)	6.6 (2.1)
	*Week 4*	5.7 (1.7)^a^	5.3 (1.4)	6.2 (2.0)
	*Week 8^†^*	5.7 (1.7)^c^	5.1 (1.2)^c^	6.5 (1.9)

CGI-S	*Baseline*	4.5 (0.7)	4.5 (0.8)	4.5 (0.7)
	*Week 4*	3.2 (1.1)^a^	2.5 (0.6)^a^	4.2 (0.8)^a^
	*Week 8^†^*	2.8 (1.1)^b,c^	2.5 (1.0)^c^	3.3 (1.1)^b,c^

QLS	*Baseline*	58.8 (20.1)	57.9 (20.9)	60.0 (19.3)
	*Week 4*	68.3 (19.1)^a^	75.1 (18.0)^a^	58.5 (16.3)
	*Week 8^†^*	67.9 (20.8)^c^	74.8 (20.0)^c^	58.1 (18.1)

*Responder status determined after 4 weeks of risperidone at a dose of 2 mg/day.

^†^Week 8 scores are on an LOCF basis for all measures.

^a^Significant change from baseline to week 4, *P* ≤ .05.

^b^Significant change from week 4 to week 8, *P* ≤ .05.

^c^Significant change from baseline to week 8, *P* ≤ .05.

BPRS: the Brief Psychiatric Rating Scale, CGI-S: Clinical Global Impression Severity Scale, QLS: Quality of Life Scale.

**Table 3:** Summary of the reports of extrapyramidal symptoms.

	Number of participants experiencing symptoms at week 4 and week 8*			Cumulative number of participants experiencing symptoms during the study^†^	

Extrapyramidal symptom	Baseline (*N* = 96)	Week 4 (*N* = 63)	Week 8 (*N* = 55)	Weeks 1–4 after baseline	Weeks 1–8 after baseline

Akathisia	3 (3.1%)	4 (6.4%)	1 (1.8%)	13	13
* Mild*	2	3	1	10	10
* Moderate*	1	1	0	3	3

Hypokinesia				2	2
* Mild*	0	0	0	1	1
* Moderate*				1	1

Rigidity	2 (2.1%)	11 (17.5%)	11 (20%)	31	34
* Mild*	2	8	10	24	26
* Moderate*	0	3	1	7	8

Tremor	3 (3.1%)	2 (3.2%)	5 (9.1%)	15	18
* Mild*	2	2	5	15	16
* Moderate*	1	0	0	0	2

Acute dystonia					
* Mild*	0	0	0	0	0
* Moderate*					

Dyskinesia					1
* Mild*	0	0	0	0	1
* Moderate*					0

*Extrapyramidal symptoms could be reported as mild, moderate, or severe; there were no reports of a severe reaction.

^†^Patients who experienced symptoms at least once during the study period.
